# Adaptive Cooperative Search Algorithm for Air Pollution Detection Using Drones

**DOI:** 10.3390/s25103216

**Published:** 2025-05-20

**Authors:** Il-kyu Ha

**Affiliations:** Department of Computer Engineering, Kyungil University, Gyeongsan 38428, Republic of Korea; ikha@kiu.kr

**Keywords:** air pollution search, drone application, air pollution detection, search algorithm, environmental drone

## Abstract

**Highlights:**

**What are the main findings?**

- Cooperative search using two drones consumes approximately 2.6 times less CPU time than a single-drone search and covers half the search distance.
- Compared to linear search, the proposed method consumed 21 times less CPU time and required 23 times less search distance.

**What is the implication of the main finding?**

- The study provides insights into drone-assisted target exploration.
- The proposed algorithm could be extended to more than two drones.

**Abstract:**

Drones are widely used in urban air pollution monitoring. Although studies have focused on single-drone applications, collaborative applications for air pollution detection are relatively underexplored. This paper presents a 3D cube-based adaptive cooperative search algorithm that allows two drones to collaborate to explore air pollution. The search space is divided into cubic regions, and each drone explores the upper or lower halves of the cubes and collects data from their vertices. The vertex with the highest measurement is selected by comparing the collected data, and an adjacent cube-shaped search area is generated for exploration. The search continues iteratively until any vertex measurement reaches a predefined threshold. An improved algorithm is also proposed to address the divergence and oscillation that occur during the search. In simulations, the proposed method consumed 21 times less CPU time and required 23 times less search distance compared to linear search. Additionally, the cooperative search method using multiple drones was more efficient than single-drone exploration in terms of the same parameters. Specifically, compared to single-drone exploration, the collaborative drone search reduced CPU time by a factor of 2.6 and search distance by approximately a factor of 2. In experiments in real-world scenarios, multiple drones equipped with the proposed algorithm successfully detected cubes containing air pollution above the threshold level. The findings serve as an important reference for research on drone-assisted target exploration, including air pollution detection.

## 1. Introduction

Drones have recently been used in a wide range of applications, including air pollution monitoring in urban areas. Air pollution monitoring involves detecting and measuring various air pollution sources in urban spaces for multiple purposes, such as reporting air pollution levels, predicting future pollution levels, and identifying specific sources of pollution. The detection of air pollution sources is particularly challenging because of their intangible, colorless, and dynamic nature [1]. Consequently, effective search algorithms tailored to these characteristics are required [2].

This study proposes a 3D cube-based adaptive cooperative search algorithm to address the challenge of monitoring air pollution in urban areas using a fleet of drones. Given the intangible and colorless nature of air pollution, tracking pollution using solely visual data captured by drone cameras is not feasible. Instead, sensors that are capable of collecting and measuring pollutants such as CO, CO_2_, PM, NO, and NO_2_ are used to trace air pollution.

The proposed algorithm focuses on identifying points in a 3D search space where pollutant levels exceed a specific threshold by using sensor measurements. To achieve precise and efficient exploration, the search space is divided into cubic regions, and multiple drones collaborate to systematically explore these cubes. Data collection points are established within each cube, and the data are gathered iteratively. By comparing the collected data, the algorithm identifies the point containing the highest pollutant concentration and generates a new cube-shaped search area centered on this point, continuing the search iteratively to locate the target air pollution sources.

Although drones have been utilized in various ways to support the implementation of smart cities, their use specifically for air pollution monitoring remains relatively limited. Research on drone-based air pollution detection is mostly centered around single-drone approaches, as detailed in Section 2 (Related Works). Studies involving collaborative exploration using multiple drones for air pollution monitoring are particularly scarce. Furthermore, only a few studies have proposed algorithms that can be practically applied in real-world environments, and even fewer have developed drone hardware capable of implementing these algorithms and verified their effectiveness through real-world experiments. Therefore, this study proposes a novel air pollution exploration algorithm based on drone collaboration, develops compatible quadcopter hardware, and conducts real-environment experiments with the algorithm onboard. The ultimate goal of this research is to enhance the practical applicability of drones for air pollution monitoring through a comprehensive approach—from algorithm development to hardware implementation and field testing.

The contributions of this study can be summarized as follows:

First, an effective air pollution search algorithm is proposed that introduces an effective method for detecting intangible and dynamic air pollution sources. The proposed 3D cube-based air pollution search algorithm identifies localized high-pollution areas according to sensor-measured pollution levels. By dividing the search space into cubic regions, the algorithm ensures systematic exploration and provides a robust framework for detecting air pollution in 3D spaces.

Second, an adaptive search approach is presented: An adaptive algorithm is proposed that dynamically adjusts the search area based on the data collected at each stage. By exploring cube-shaped spaces and focusing on the points having the highest pollution levels, the algorithm efficiently identifies areas with high pollutant concentrations.

Third, a dynamic space adjustment is used: The algorithm enables dynamic adjustment of the search space. Instead of being restricted to a predefined search area, the algorithm expands or modifies the search region based on the collected data, thereby allowing efficient exploration, even as the search space grows, for as long as the drones’ energy resources permit.

Fourth, a collaborative multi-drone search is carried out: A collaborative method for multi-drone exploration is proposed. By dividing the search space between multiple drones that conduct searches in parallel, the algorithm reduces redundancy, increases search speed, minimizes drone battery consumption, and extends the overall duration of the mission. This collaboration enables the swift and accurate detection of locations of highly concentrated pollution within the cubic search space, providing an effective solution for air pollution monitoring.

In Section 2, prior studies on UAV-based target search and air pollution monitoring are explored, highlighting the distinguishing features of this work. Section 3 presents the justification for using a cubic search space and details the design of a cooperative air pollution search algorithm based on multiple UAVs operating within this structure. Section 4 describes the implementation of numerical simulations in MATLAB (R2015a) to evaluate the performance of the proposed algorithm, followed by the development of quadcopter-type UAV hardware for validation in real-world environments. The effectiveness of the algorithm is demonstrated through these practical experiments. In Section 5, we discuss the limitations of the proposed algorithm and hardware implementation, potential issues encountered during development, and the algorithm’s scalability. Finally, Section 6 concludes the study with a summary of findings and outlines directions for future research.

## 2. Related Works

Airborne air pollution can be considered to be a type of search target for drones. Many drone applications achieve the fundamental objective of detecting targets on the ground or in the air, which enables their use in various fields. Therefore, research on target detection, target tracking, and monitoring with the use of drones can be classified as listed in Table 1. These classes include target detection, mobile target tracking, monitoring and various purposes, and air pollution detection.

Target detection focuses on detecting targets on the ground or in the air. A general approach to understanding this field is to review survey studies that analyze the existing research. For example, Adoni et al. [3] discussed the challenges and communication problems involved in implementing autonomous multiple unmanned aerial vehicle (UAV) systems. Their study emphasized that building cooperative drone systems without human intervention is highly complex and requires some consideration of UAV types, mission complexity, communication architectures, and routing protocols. It also provides a comparative analysis of existing studies and offers guidance for selecting appropriate communication architectures.

Iftikhar et al. [4] highlighted the role of target detection in smart cities, such as traffic congestion management. Object detection in smart city environments is challenging because of factors such as the movement of people or objects, and the small sizes of objects. This study analyzed various deep learning approaches to improve detection accuracy, reduce computational costs, and optimize system design.

Several studies have focused on single-drone target detection. For example, Zhang [5] and Wang et al. [6] explored methods for detecting objects in drone-captured images. Zhang [5] explained that images captured by drones differ from ground-level images in terms of object size, density, occlusion, and lighting conditions. To address these challenges, their study proposed an object detection algorithm based on the YOLOv8 model, which was designed for more effective detection on drone imagery. Wang et al. [6] further stated that UAVs capture images from high altitudes, leading to smaller object sizes and the presence of multiple objects, necessitating specialized target detection algorithms. Their study introduced a YOLOv8-based UAV target detection algorithm tailored to these conditions.

Recent studies have explored collaborative drone target detection and investigated applications in which multiple drones cooperate for target detection. Several notable studies, such as those by Minaeian et al. [7], Zhu et al. [8], Stasinchuk et al. [9], and Alotaibi et al. [10], have contributed to this field. Minaeian et al. [7] examined a method for the tracking and control of crowds in border areas through the cooperation of UAVs and multiple unmanned ground vehicles (UGVs). Zhu et al. [8] proposed a distributed online heuristic strategy to solve the problem of multi-UAV collaborative coverage. Stasinchuk et al. [9] addressed how a team of cooperating autonomous aircrafts could safely intercept multiple intruder UAVs. Alotaibi et al. [10] explored how a team of multiple unmanned aerial vehicles could efficiently complete search and rescue missions and save the maximum number of people.

The field of mobile target tracking in target detection research focuses on detecting or tracking targets that move dynamically. Alhafnawi et al. [11] conducted a valuable study for researchers seeking to understand trends in drone-based target tracking. The key design challenges for target-tracking drones were identified, and ideas for addressing them were introduced. Among the studies on mobile target tracking, some have focused on applications that use a single drone, such as those by Wang et al. [12] and Zheng et al. [13]. Wang et al. [12] proposed a framework for tracking moving ground targets using a fixed-wing UAV. Zheng et al. [13] presented a method involving collaboration between humans and UAVs for tracking escaped criminals by introducing a hybrid evolutionary algorithm to explore the search space efficiently.

Research on cooperative applications involving multiple drones in mobile target tracking includes studies by Nathan et al. [14], Cimino et al. [15], Opromolla et al. [16], Hentati et al. [17], Zhou et al. [18], SaaDaoui et al. [19], and Dai et al. [20]. Nathan et al. [14] investigated how autonomous drones detected and tracked concealed targets in densely forested areas. Cimino et al. [15] explored the coordination of UAVs using deep learning methods to detect and track dispersed targets in various search environments. Opromolla et al. [16] studied how drones within a swarm visually recognized other drones using a machine vision approach based on the You Only Look Once (YOLO) module. Hentati et al. [17] proposed a cooperative UAV framework for tracking moving ground objects. Zhou et al. [18] examined a method in which multiple UAVs collaborate to track multiple intruders that are infiltrating military security facilities. SaaDaoui et al. [19] introduced a dynamic UAV formation and target search model using a cooperative particle swarm optimization (PSO) algorithm to track moving ground targets. Dai et al. [20] presented a 3D collaborative trajectory optimization (CTO) approach for tracking multiple UAVs in air. All of these studies contribute to the broader field of ground and aerial target tracking using UAVs.

Third, studies have been conducted on the use of drones for monitoring and other applications. Most of these studies have focused on drone applications for monitoring urban traffic, environmental conditions, and disasters. Some key studies have provided insight into research trends in this field. Alsamhi et al. [21] investigated and analyzed recent research on drone collaboration and Internet of Things (IoT) applications for the realization of smart cities. Popescu et al. [22] examined and analyzed application cases for integrated systems based on wireless sensor networks (WSNs) and UAVs. Gohari et al. [23] conducted a literature review and identified seven research areas related to surveillance drone usage in smart cities. Chmaj et al. [24] explored case studies where multiple UAVs were used in distributed processing for surveillance and environmental monitoring. Eche et al. [25] provided a systematic analysis of the literature related to forest health monitoring (FHM), offering insights into research trends, required technologies, and the advantages and disadvantages of various approaches. Alsamhi et al. [26] analyzed the key technologies, data collection methods, and energy efficiency requirements for green IoT and smart cities by performing a review of the relevant research literature.

Among the studies that have been conducted on monitoring and other applications, some have focused on single-drone utilization, such as those by Langhammer [27] and Oltmanns et al. [28]. Langhammer [27] explored the use of UAVs to monitor river restoration in urban environments, and Oltmanns et al. [27] introduced UAV applications for soil erosion monitoring. Studies focused on drone collaboration and multi-drone applications include those of Fu et al. [29], Elloumi et al. [30], Liao et al. [31], and Pan et al. [32]. Fu et al. [29] investigated energy-efficient methods for allocating search spaces and avoiding collisions when multiple UAVs are tracking aerial targets. Elloumi et al. [30] studied a traffic monitoring system that used multiple UAVs to track vehicles. Liao et al. [31] examined efficient and stable data transmission between drones for environmental monitoring applications. Pan et al. [14] focused on communication protocols and platforms that enabled UAVs to collect and transmit monitoring data from disaster-affected areas.

Fourth, studies related to air pollution detection using drones have focused on atmospheric pollution monitoring. To understand the characteristics and trends of these studies, researchers such as Motlagh et al. [1], Fascista [2], Villa et al. [32], Javaid et al. [33], Ye and Geng [34], Zhou et al. [35], and Motlagh et al. [36] have provided valuable insights. Motlagh et al. [1] presented the key challenges in UAV-based air quality monitoring by performing a literature review and suggested a research roadmap for future studies. Fascista [2] discussed the available solutions and limitations of large-scale environmental monitoring using UAVs. Villa et al. [32] reviewed UAV-based air pollution and emission monitoring studies, analyzed current technological advancements, and identified unresolved challenges. Javaid et al. [33] introduced communication and control methods and application cases in cooperative multi-UAV systems through a literature survey. Geng [34] explained pollution monitoring principles and methodologies for monitoring port environments using UAVs. Zhou et al. [35] categorized UAV swarm intelligence research into five hierarchical levels and discussed the research trends at each level. Motlagh et al. [36] addressed urban-scale air pollution monitoring using UAVs and discussed the vertical distribution of urban air pollution, monitoring requirements, and research challenges.

Among the studies on air pollution detection, various research cases have focused on single-drone applications. Araujo et al. [37] analyzed the performance of air pollution sensors mounted on UAVs under different flight conditions. Alvear et al. [38] proposed a method for mapping air pollution by using a single drone to focus on areas with high pollutant concentrations. Gu et al. [39] introduced a modular UAV-based platform capable of monitoring various air pollutants in real time. Zhou et al. [40] considered the aerodynamics of a six-rotor UAV equipped with air pollution sensors and presented case studies on particulate matter (PM) measurements. Alvear et al. [41] examined the optimal search space size and accuracy of energy-efficient air pollution monitoring by multirotor UAVs. Vega et al. [42] proposed an air pollution monitoring system using UAVs and demonstrated data transmission, information processing, and a graphical user interface (GUI)-based monitoring case study. Pochwala et al. [43] presented a low-cost air pollution sensor system mounted on UAVs and took vertical PM measurements during UAV vertical flights.

Compared with single-drone applications, collaborative UAV applications for air pollution detection are relatively rare. Examples of such studies include those by Liu et al. [44], Fu et al. [45], Bolla et al. [46], Naula et al. [47], and Liu et al. [44], who proposed an AI model that analyzed air pollution haze images captured by a UAV swarm consisting of server UAVs and client UAVs to predict air quality indices. This study demonstrated the efficiency of the model through multiple experiments. Fu et al. [45] presented a research case in which multiple UAVs tracked the dispersion paths of hazardous gas. Bolla et al. [46] proposed a vertical take-off and landing UAV that was capable of studying air quality at different altitudes, and they considered airflow effects from the UAV propellers to determine the vertical drone deployment. Naula et al. [47] introduced an algorithm to locate air pollution sources using UAVs. The algorithm employed a heuristic method to track the gradient of pollutant concentration and used a probabilistic approach to search promising areas. However, this study was conducted in a controlled indoor environment rather than in a real-world environment.

External factors such as wind may have a potential impact on the accuracy of air pollution measurements when using drones. Therefore, this study reviews prior research addressing the influence of wind on UAV operation and pollutant detection.

Foundational studies on the effects of wind on UAV flight can be found in the works of Wang et al. [48] and Wang et al. [49]. Wang et al. [48] discusses fundamental challenges related to wind in low-altitude UAV operations. The study categorizes different wind types and their characteristics, presents mathematical models, and explains how UAVs behave in windy environments in terms of velocity, force, and energy consumption. Wang et al. [49] highlights how small UAVs are highly sensitive to wind disturbances, which can impair their ability to maintain altitude, speed, and position. The authors conduct a detailed analysis of wind mechanisms, such as wind shear and turbulence, and propose strategies to mitigate their impact on UAVs.

Additional studies have explored case-specific effects of wind on UAV operations. For instance, Yang et al. [50] analyze how sudden changes in wind direction and intensity affect UAV performance. Through numerical simulations, they evaluate the aerodynamic performance of UAV wing profiles under various wind shear conditions, concluding that positive-gradient shear is favorable for cruise flight, while negative-gradient shear benefits takeoff and landing. Yang et al. [51] propose a path-following algorithm for fixed-wing UAVs and assess how wind impacts path-tracking performance. Their results emphasize the significant aerodynamic influence of wind on fixed-wing UAVs and the need to account for wind in navigation algorithms.

Prudden et al. [52] investigate small UAVs used for aerial wind measurement, discussing practical considerations and limitations. Their study demonstrates the feasibility of measuring average wind speed and turbulence intensity at various altitudes while hovering. Gianfelice et al. [53] examine how urban turbulence and localized high-velocity wind corridors caused by tall buildings affect UAV flight, employing Computational Fluid Dynamics (CFD) techniques for in-depth analysis.

Although studies specifically addressing the impact of wind on air pollution detection are limited, several notable works exist. Lee et al. [54] analyze how urban obstacles such as buildings and noise barriers affect air quality measurements taken by drones in city environments, with particular focus on the influence of wind direction on pollutant concentration. Araujo et al. [37] conduct experimental studies using drones equipped with sensors to measure air pollutants under various conditions. Their results show that under low wind speeds, differences between flight patterns are minimal, whereas under high wind speeds, flight patterns significantly affect NO_2_ concentration measurements. Pochwala et al. [43] employ UAVs to explore vertical distributions of air pollution while using wind speed as a measurement parameter, although they do not examine how wind strength alters pollution readings collected by drones.

These studies collectively indicate that wind strength can meaningfully impact drone-based air pollution detection and thus must be considered in future research and algorithm development.

**Table 1:** Classification of research on drone-based target detection, target tracking, and monitoring.

Drone	Target Detection	Mobile Target Tracking	Air Pollution Detection	Monitoring and Various Purposes	Impact of Wind
Single-Drone Operation	[5,6]	[12,13]	[37,38,39,40,41,42,43]	[27,28]	[37,43,50,51,52,53,54]
Cooperative Flight	[7,8,9,10]	[14,15,16,17,18,19,20]	[44,45,46,47]	[29,30,31,32]	
Survey	[3,4]	[11]	[1,2,32,33,34,35,36]	[21,22,23,24,25,26]	[48,49]

In addition to the studies mentioned above, the following works also provide valuable insights for designing target search algorithms.

Czyz et al. [55] investigate the deformation of unmanned aircraft fuselages under various flight conditions and explore a method to reduce the mass and increase the balance coefficient of the UAV by modifying the fuselage geometry.

Ambrozkiewicz et al. [56] address a method for quantitatively measuring and detecting damage to the electric motors of unmanned aerial vehicles (UAVs). In particular, they present a detailed approach for identifying and quantifying different levels of motor damage in an octocopter—a drone with eight arms—by systematically varying the damage to one of its electric motors.

Kotarski et al. [57] discuss the educational application of unmanned aerial vehicle (UAV) platforms. In particular, they focus on how to incorporate UAV hardware components, software elements, mathematical modeling, and flight-based applications into undergraduate curricula.

Our proposed 3D cube-based drone collaborative exploration algorithm offers several key advantages over existing algorithms: The first advantage is its enhanced search efficiency through structured space division: Although many existing studies determine UAV search paths heuristically or by using sensors, our 3D cube-based approach structures the search space systematically and enables efficient drone collaboration. This minimizes redundant searches and optimizes the search trajectories. The second advantage is faster detection of high-pollution areas through collaboration: Unlike single-drone approaches [37,38,39,40,41], our method allows multiple drones to collaborate within cubic units and to identify target areas more quickly. Specifically, it is designed to resolve problems of divergence and oscillatory behavior, thereby ensuring stable and effective exploration. The third advantage is precision search that considers both vertical and horizontal expansions: Although some existing studies [36,47] have suggested vertical exploration, our algorithm leverages a 3D cube structure to balance both vertical and horizontal exploration. This allows for a more precise analysis of the air pollution distribution in both aerial and ground-level regions. The fourth advantage of the algorithm is its adaptive search capability: Many UAV-based search studies follow fixed search paths, whereas our algorithm dynamically adjusts its search patterns according to the pollution concentration. This enables the algorithm to focus on high-concentration areas while minimizing its exploration in low-priority regions, thereby reducing search costs. The fifth advantage of the algorithm is that it is applicable to diverse environments: Some existing studies [44,45,46] have been validated only in limited environments (e.g., indoor experiments and constrained spaces). In contrast, our algorithm is designed to allow multiple drones to collaborate effectively in real-world conditions, enabling deployment in urban areas, industrial zones, and forests.

## 3. Three-Dimensional Cube-Based Adaptive Cooperative Search Algorithm

This section explains the advantages of the cube-based exploration method in a 3D space and proposes a 3D cube-based adaptive cooperative search algorithm, in which two drones collaborate to explore the three-dimensional space efficiently.

### 3.1. Comparison of Exploration Methods in a 3D Space

**Figure 1:**
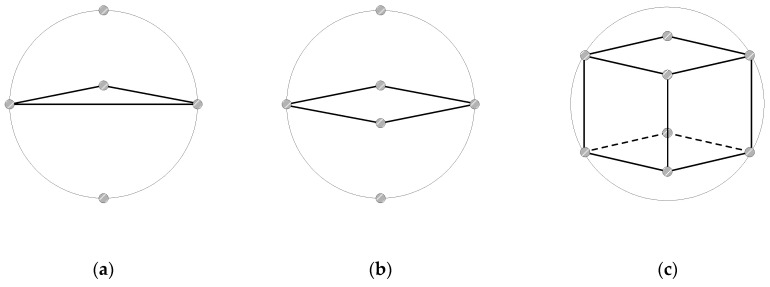
Different data collection strategies within a spherical space. (**a**) Triangular-based data collection, (**b**) Square-based data collection, (**c**) Cube-based data collection.

To compare different data collection strategies within a spherical space, we consider three geometrical approaches: cube-, square-, and triangle-based vertex arrangements, as shown in Figure 1. Each of these methods involves collecting data from specific vertices within a sphere, and they vary in terms of the number of vertices sampled and the spatial coverage.

Triangular-Based Data Collection: This method gathers data from five points: three vertices forming a triangle in a single plane, as well as from the topmost and bottommost points of the sphere. Although this approach is simple and energy efficient, it provides limited spatial coverage, primarily in the horizontal plane. The vertical dimension is only partially covered by the top and bottom points, which results in less comprehensive 3D coverage than other strategies. It is best suited for scenarios where minimizing the number of data collection points is crucial, such as rapid scanning or energy-constrained operations.

Square-Based Data Collection: With this method, data are collected from six points: four vertices that form a square in a single plane and the topmost and bottommost points of the sphere. This provides a better spatial distribution than does the triangular method, with improved symmetry along the vertical axis. However, the horizontal coverage remains confined to one plane, which leaves gaps in the full 3D distribution. This is particularly useful for applications where vertical data collection is a priority, such as atmospheric monitoring, while maintaining a relatively simple setup with fewer data points.

Cube-Based Data Collection: This method collects data from eight vertices of a cube inscribed within a sphere, and it offers highly symmetric and evenly distributed spatial coverage across all three dimensions. The cubic structure ensures comprehensive exploration of the spherical space, making it ideal for detailed 3D mapping. However, movement between vertices may require longer distances, which can be challenging in obstacle-rich environments. This approach is best suited for applications that require high-precision data collection with extensive three-dimensional coverage.

The cube-based data collection method is particularly well-suited for applications such as air pollution monitoring, where comprehensive and detailed spatial coverage is crucial. This method provides an evenly distributed set of data points in three-dimensional space. Each vertex is positioned at the intersection of the x-, y-, and z-axes, thereby ensuring a balanced distribution that covers the entire volume under investigation.

The validity and advantages of using a cubic search structure can be summarized as follows. First, it allows for maximized utilization of the search space. Since air pollution may exist anywhere within the environment, it is important to explore the space as widely as possible to increase the likelihood of detection. The cubic approach enables broad and systematic exploration of the given area.

Second, it provides efficient coverage of data collection points. By collecting data at each vertex of the cube, the method ensures spatially uniform sampling, which enhances the probability of detecting locally concentrated pollutants.

Third, the spatial symmetry of the cubic structure makes it well-suited for cooperative multi-drone exploration. Because of its symmetrical nature, the space can be evenly divided among multiple drones, enabling effective vertical or horizontal partitioning for parallel search operations.

Fourth, the use of linear flight paths simplifies drone control. Compared to curved trajectories, linear flights are easier to control and plan, which contributes to simpler path planning and algorithm implementation.

Therefore, we propose an adaptive method for air pollution monitoring that uses a cube-based exploration algorithm, in which multiple drones (in this case, two drones) collaborate to explore a cubic search space. This approach is adaptive because the search area is determined from the results of previous explorations, thereby allowing for dynamic adjustments and more efficient data collection. By focusing on the areas of interest identified during the early stages of exploration, this method ensures comprehensive coverage while optimizing the search process.

### 3.2. Design of the 3D Cube-Based Adaptive Cooperative Search Algorithm

**Figure 2:**
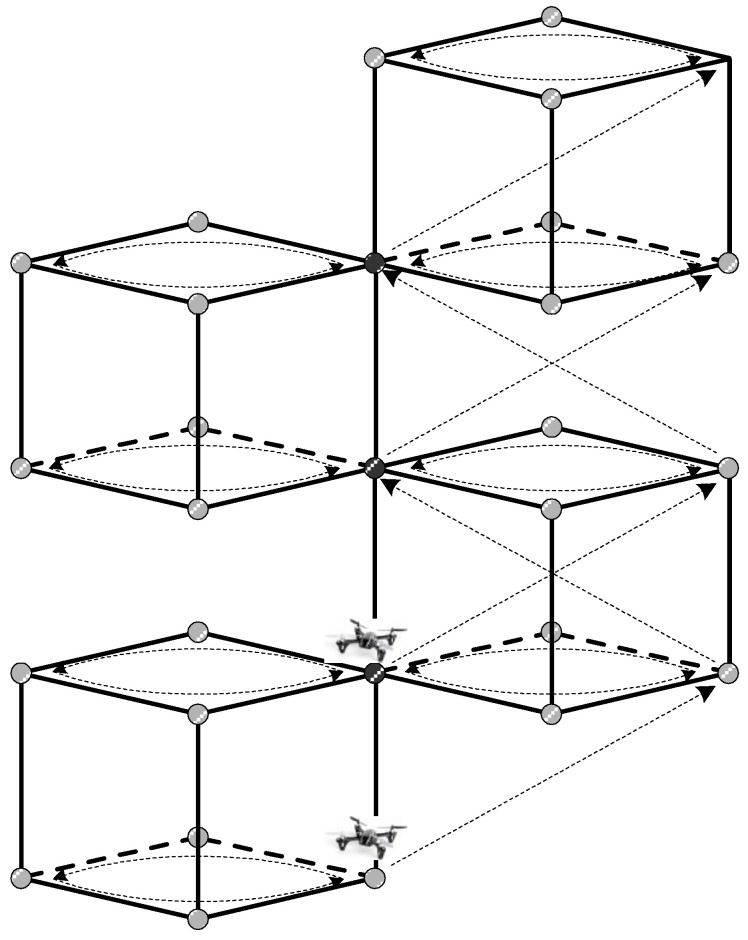
Basic concept of 3D cube-based cooperative exploration.

Figure 2 shows the search process for the proposed algorithm, which operates as follows. First, the cubic space is divided into regions, with each drone assigned to explore a specific area. For example, in the case of two drones, one explores the upper portion of the cube, whereas the other explores the lower portion. Second, each drone systematically visits the data collection points within its assigned cubic region and gathers air pollution data. To ensure accuracy, each cubic search space is explored multiple times (n iterations), which allows for repeated data collection at each point.

Third, after each exploration round, the collected data are examined to determine whether any values exceed a predefined threshold. If any data points are detected above the threshold, the exploration is terminated. If no such data are found, the algorithm proceeds to the next step. Fourth, based on previous exploration results, the adjacent search area is determined, and the exploration region is expanded. Specifically, the average value of the data collected at each of the eight vertices of the cubic space is calculated, and the vertex having the highest value is selected. A new spherical and cubic search space is then generated with the highest point as a reference. The exploration continues according to the method described in step one. This process is repeated until data exceeding the threshold are obtained.

This adaptive exploration algorithm dynamically adjusts the search region according to prior results, ensuring that the drones focus on areas of interest. This method optimizes data collection while maintaining comprehensive coverage by expanding the search space only when required. This approach is particularly effective for air pollution monitoring, where the spatial distribution of pollutants can vary, and an adaptive search strategy can help to identify areas with high pollution levels.

The proposed exploration algorithm can be formalized as follows:

- Notation

Let *C_i_* denote the *i*-th cube in the search space with 8 vertices, *V_i_* = {*v_i_*_1_,*v_i_*_2_, …, *v_i_*_8_}.

Let *D*1 and *D*2 represent two drones that explore the upper and lower regions of each cube, respectively.

Let *T* denote the threshold value for pollution detection.

Let $m_{v_{ij}}^{\left(k\right)}$ denote the measurement collected at vertex *v_ij_* of cube *C_i_* during the *k*-th exploration iteration.

Let *n* represent the number of iterations per cube for data collection.

Let ${\bar{m}}_{v_{ij}}$ denote the average measurement at vertex *v_ij_* over *n* iterations.

(1) $${\bar{m}}_{v_{ij}}=\frac{1}{n}\;\sum_{k=1}^{n}m_{v_{ij}}^{\left(k\right)},\;\;\forall v_{ij}\;\in \;V_{i}$$

Let $v_{max}$ be the vertex with the maximum average measurement in cube *C_i_*.

(2) $$v_{max}=arg\;\underset{v_{\mathrm{ij}}}{\max}\left({\bar{m}}_{v_{ij}}\right),\;\;\forall v_{ij}\;\in \;V_{i}\;\;$$

Let *C_i+_*_1_ be the next cube selected for exploration.

Below are the detailed steps of the algorithm.

- Step 1: Initialization
  1. Set the initial cube search count *n* = 0.
  2. Set the pollution detection threshold *T*.
  3. Initialize target_found = False.
  4. Drones *D*1 and *D*2 start from the ground and ascend to the search altitude.
- Step 2: Cooperative Exploration and Data Collection

For each cube *C_i_*,

1. Drones *D1* and *D2* reach the cube’s starting position.
2. Each drone explores a subset of vertices:

*D*1 explores the upper vertices: $V_{i}^{top\;}=\{v_{i1},v_{i2},v_{i3},v_{i4}\}$.

*D*2 explores the upper vertices: $V_{i}^{bottom\;}=\{v_{i5},v_{i6},v_{i7},v_{i8}\}$.

3. Each drone performs *n* iterations, measuring pollution levels at each assigned vertex.
4. The average measurement at each vertex is computed as:

(3) $${\bar{m}}_{v_{ij}}=\frac{1}{n}\;\sum_{k=1}^{n}m_{v_{ij}}^{\left(k\right)}$$

5. The maximum measured value in the cube is determined as:

(4) $$m_{max}=\underset{v_{\mathrm{ij}}\in \;V_{i}}{\max}\left({\bar{m}}_{v_{ij}}\right)\;$$

- Step 3: Threshold Check and Search Termination

If $m_{max}\geq T$, the search terminates, and the drones return to the starting point:

target_found=True

Otherwise, the algorithm proceeds to the next step.

- Step 4: Adaptive Search for the Next Cube

1. Identify the adjacent cube with the highest neighbor value:

(5) $$v_{max}=arg\;\underset{v_{\mathrm{ij}}\in \;V_{i}}{\max}\left({\bar{m}}_{v_{ij}}\right)\;$$

2. If a neighboring cube *C_i_*_+1_ contains a vertex *v_max_* such that *m_max_* < *m_neighbor_*, then the maximum value is updated:

$$m_{max}=m_{neighbor}$$

3. If $m_{max}\geq T$, terminate search.

4. Otherwise, the drones move to the adjacent cube *C_i_*_+1_ and continue.

Algorithm 1 shows the cube-based adaptive cooperative search algorithm.
**Algorithm 1.** 3D Cube-Based Adaptive Cooperative Search Algorithm1initialize n (cube search count)2initialize th (search threshold value)3target_found = False4Drone 1 and Drone 2 begin their exploration from a ground starting point5Drones reach the search altitude6**while** (target_found ! = True)7 Drones arrive at cube starting point8 **while** (search_count >= n)9  Drones explore the cube’s vertices, collect data, and transmit it10  Search_count++11 
**end-while**
12 Processing collected data and calculating maximum values13 **if** (maximum_value >= th)14  **while** (next maximum_value)15   **while** (next neighbor cube) 16    **if** (maximum_value < neighbor_value) 17     maximum_value = neighbor_value 18    
**end-if**
19   
**end-while**
20   **if** (maximum_value >= target_value)21    target_found = True 22    Drones move to the starting point23   
**else**
24    Next maximum_value25   
**end-if**
26  
**end-while**
27 
**else**
28  Drones move to the cube adjacent to the maximum value vertex29 
**end-if**
30**end-while**

At the beginning of an iteration of the algorithm, the search count (*n*) and the threshold value (*th*) for target detection are initialized. The flag target found is set to False to indicate that the target has not yet been located. The two drones begin their mission from a designated ground position and ascend to their operational altitude. From there, they navigate to the starting position of the first cube in the search space.

Once positioned at the cube, the drones begin their exploration by systematically collecting data from its vertices. Each measurement is recorded and transmitted, and this process is repeated for *n* iterations to enhance accuracy. After the data are collected, the algorithm processes the gathered information and determines the maximum recorded value within the cube. If this value exceeds the threshold *th*, the algorithm conducts a secondary search among the neighboring cubes to find a value greater than the threshold. The algorithm compares the highest recorded value within the current cube to those in adjacent cubes, and the maximum value is updated accordingly.

If the maximum value in any cube reaches or exceeds the defined target value, the target_found flag is set to True, indicating that the search has been successful. The drones then return to their starting positions to complete the search process. If the target value is not detected, the drones move to the neighboring cube that contains the vertex at which the highest recorded measurement was taken and continue the exploration process. This iterative search strategy ensures that drones systematically navigate the environment and focus on areas with the highest potential target values.

One key feature of this algorithm is its cooperative exploration approach, in which two drones work together to cover a defined cubic search space. Additionally, its adaptive search strategy dynamically adjusts the search direction based on data collected in real time. This ensures efficient data processing, allowing drones to effectively identify and prioritize areas of air pollution with high concentration. The search process terminates immediately when the target value is detected, which optimizes both time and resource utilization.

By iteratively expanding the search to the adjacent cubes having the highest potential values, the algorithm ensures thorough and efficient exploration of 3D space. This method is particularly useful for applications such as environmental monitoring, pollution detection, and other scenarios that require systematic 3D spatial search capabilities.

The problems of divergence and oscillatory behavior were carefully considered when this algorithm was designed. This approach ensures that the mission can be terminated once air pollution exceeds a certain threshold. However, to identify the 3D cubic cell with the highest value, it is crucial to address these two potential issues.

A divergence problem may arise when no 3D cube cell having the same measurement values is considered after all eight vertices are explored. If one of these equally valued cells is selected, and the search is extended to an adjacent 3D cube cell, the search process may diverge, leading to inefficient exploration. To mitigate this problem, all adjacent 3D cube cells that share the same measurement value should be considered when the search space is expanded.

An oscillatory behavior problem occurs because the search expansion is limited to the eight vertex-connected directions of the current 3D cube cell. This restriction leads to a failure to consider expansions along the same plane or in the vertical (upward and downward) directions, causing the search to oscillate between specific cube cells. To address this issue, before the search is expanded in the eight vertex-connected directions, the algorithm should first consider exploring the cells in the planar and vertical directions relative to the current 3D cube cell.

When two drones are used to cooperatively explore a cubic space in air, several challenges arise, particularly those related to collision avoidance and coordination. The potential issues and solutions addressed in this study are as follows.

First, the flight collision risk problem: Because drones explore the same cubic space, they may risk collision, especially if their paths cross near the edges or vertices of the cube. This problem arises when two drones move after exploring one cube-shaped space and then generate another cube-shaped space based on the highest point. This issue can be resolved by allowing the drone that explored the region where the highest point was found, either in the upper or lower parts of the cube, to move first. For example, when the highest point appears in the upper part of a cube, the drone that explored the upper section moves first to the starting point of the newly formed cube. After a certain time interval, the drone that explored the lower part follows the first drone and moves into the new cube exploration area. Additionally, the two drones are designed to exchange information and maintain a certain distance (approximately 10 m) during their flight to prevent collisions.

Communication latency or failure: While data are collected in a cube-shaped exploration space, the two drones exchange location information with the server and collect data. If communication delays or failures occur, it may be difficult to accurately determine the drones’ positions, which could lead to collisions or other problems. To address this, this study was designed so that the drones remain in their current positions and wait until normal communication is restored. Additionally, when the two drones send collected data to the server, which occurs periodically, communication delays or failures can hinder the reception and processing of data, making it difficult to proceed to the next step. To resolve this issue, this study assigns a drone ID to the collected data and adjusts the data transmission intervals to ensure that the server receives data from both drones consistently.

Third, the problem is limited by energy management and the exploration area: drones have a limited battery life, which means that they must complete their mission within a constrained timeframe. If the threshold value is not detected within the lifetime of the battery, the drones may experience power failure or other problems. Additionally, excessive expansion of the exploration area may lead to communication difficulties with the server or make it challenging for the drones to return to their starting point. To address these concerns, this study restricts the exploration flight time based on battery life and limits the exploration area to a defined range, thereby ensuring that the mission can be completed without power- or communication-related issues.

## 4. Performance Analysis

A performance analysis of the proposed 3D cube-based adaptive cooperative search algorithm was conducted. First, various simulations were performed to demonstrate that the proposed search algorithm is more efficient than other methods. To evaluate the performance of the proposed algorithm in a real-world environment, a quadcopter drone equipped with the proposed algorithm was developed and tested in real-world experiments. The results were then analyzed.

The simulation and experiment are conducted as follows. The simulation described in Section 4.1 refers to a numerical performance analysis of the proposed algorithm using MATLAB (R2015a). The experiment described in Section 4.3 refers to a real-world performance evaluation, where the proposed algorithm is implemented on an actual drone developed for this study.

### 4.1. Simulation

**Figure 3:**
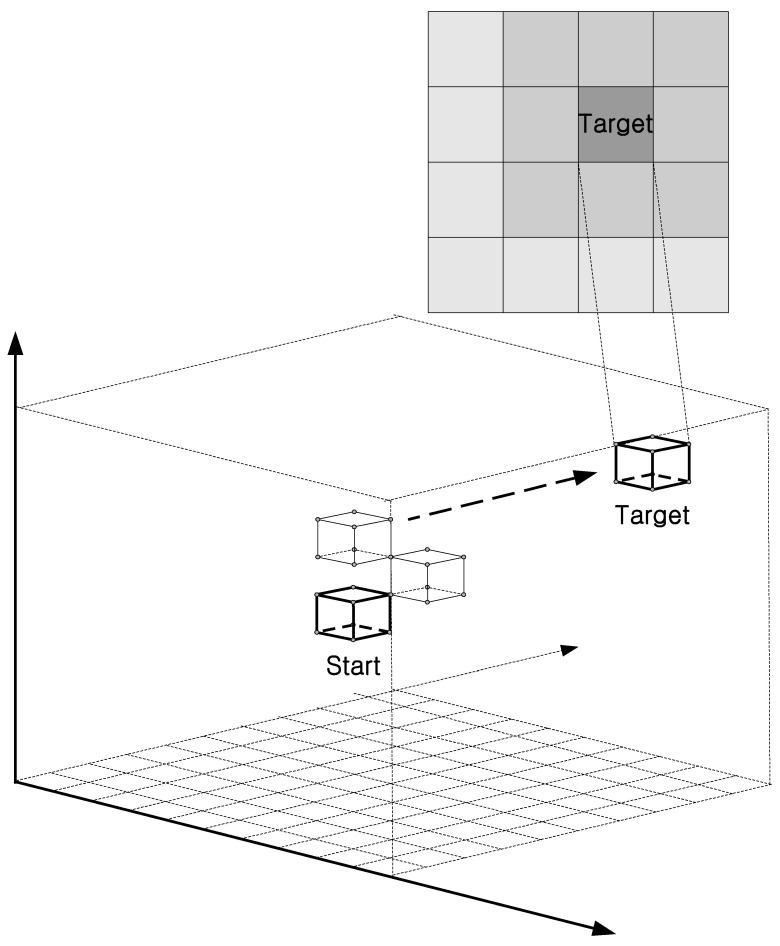
Simulation environment.

Various simulations were conducted to analyze the performance of the proposed 3D cube-based adaptive cooperative search algorithm. Table 2 lists the simulation conditions. The search space was a three-dimensional area consisting of 10 cells along the *x*-, *y*-, and *z*-axes. A single air pollution target with the highest concentration was randomly determined at the beginning of each round. Depending on the simulation mode, one or two drones cooperated in the search. The maximum number of cells that could be explored per round was set to 1000. Figure 3 illustrates the simulation environment. The drone begins its search from the center of the three-dimensional search space and explores a randomly determined air pollution target in each round. It is assumed that the target contains the highest air pollution concentration and that the surrounding pollution levels gradually decrease.

In the simulation, four search methods were compared: (1) the proposed 3D cube-based search algorithm executed by a single drone (CubeSearch (D1)), (2) the proposed algorithm executed cooperatively by two drones (CubeSearch (D1+D2)), (3) a linear search method that explores the search space sequentially (Linear), and (4) the proposed algorithm applied without considering divergence or oscillation (Divergence (D1)).

**Figure 4:**
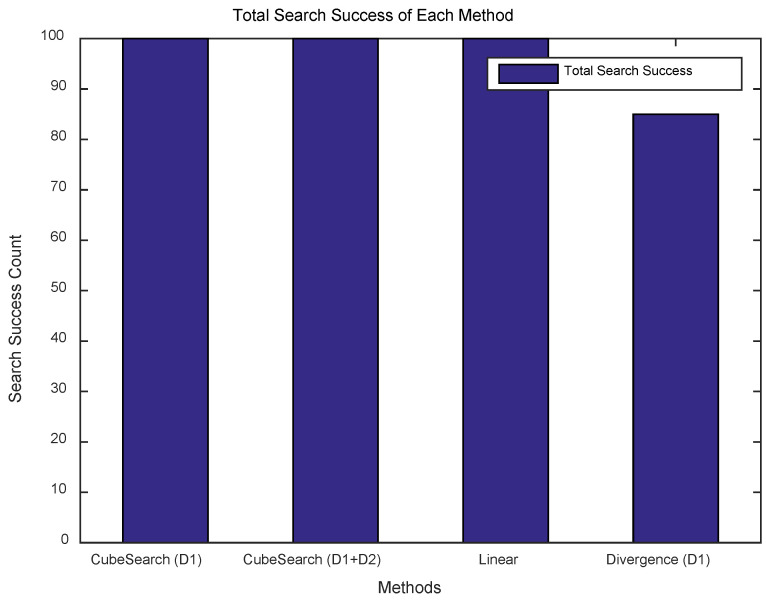
Total search success for each method.

The results of conducting searches over 100 rounds with randomly generated targets in the search area are shown in Figure 4. As illustrated in Figure 4, CubeSearch (D1), CubeSearch (D1+D2), and the linear method achieved a 100% search success rate, whereas the divergence (D1) method exhibited a success rate of only 85%, owing to divergence and oscillation.

**Figure 5:**
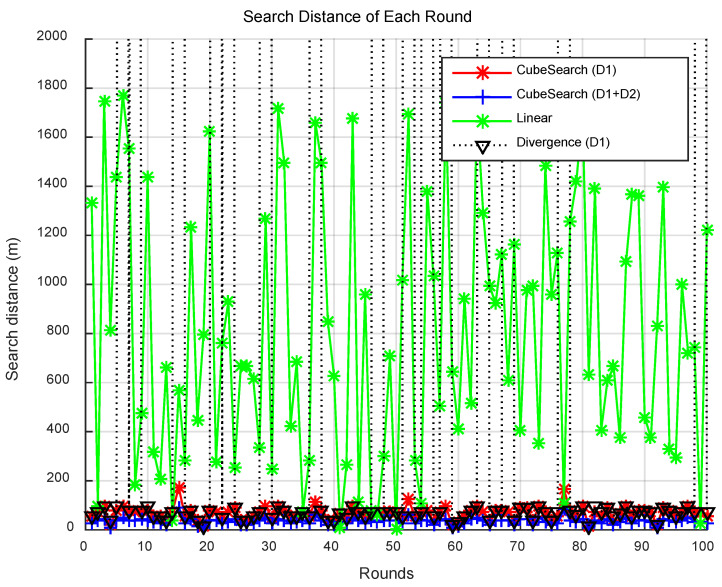
Search distance for each round.

**Figure 6:**
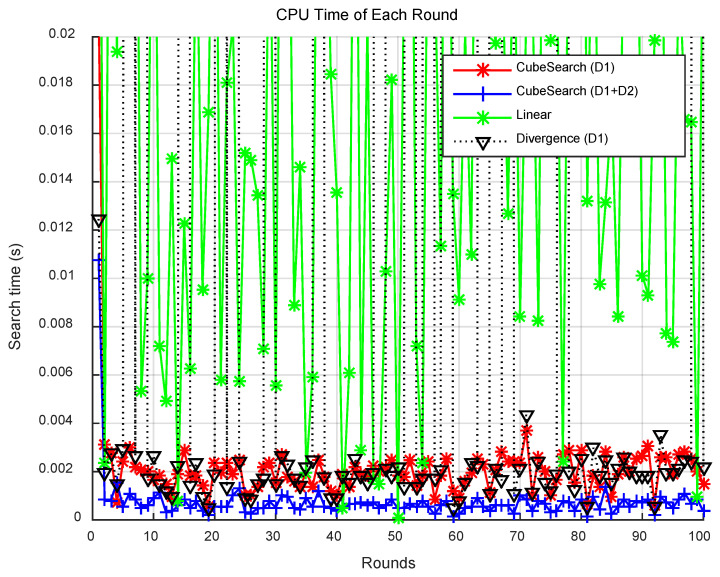
CPU time for each round.

**Figure 7:**
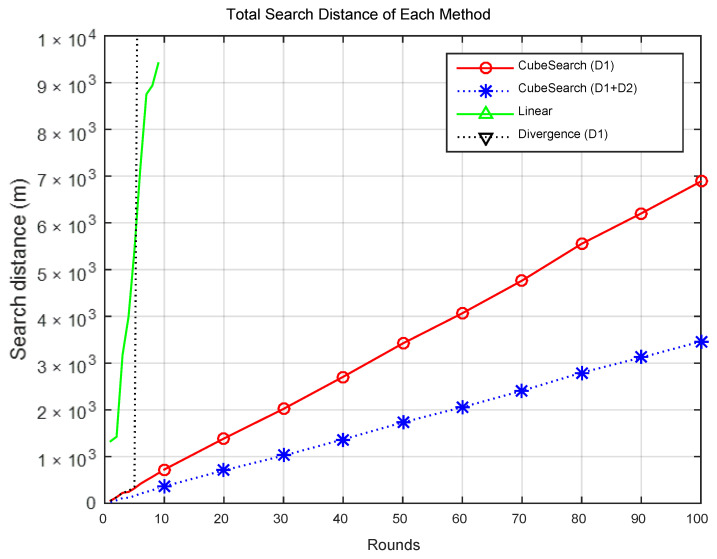
Total search distance for each round.

**Figure 8:**
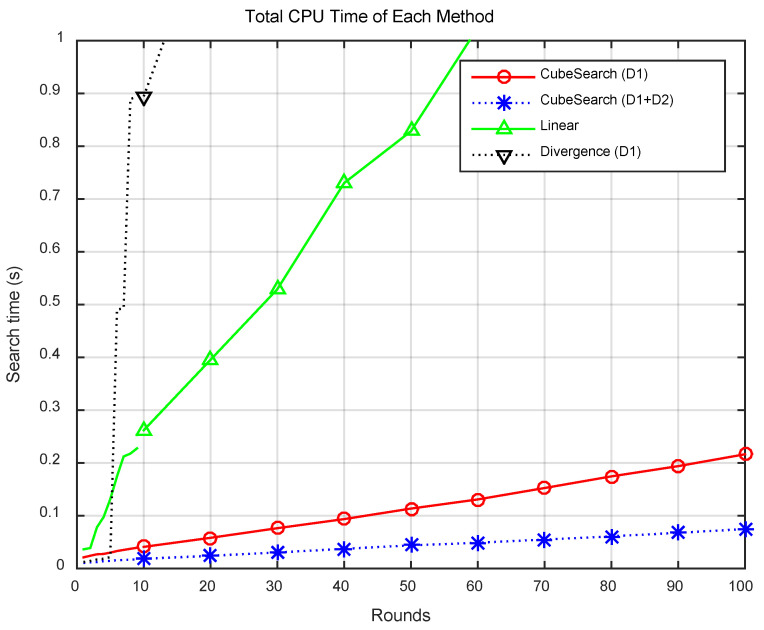
Total CPU time for each round.

Figure 5 presents the search distance per round for each method, and Figure 6 displays the CPU time per round. Compared with the CubeSearch methods, the Linear and Divergence methods demonstrated relatively higher search distances and longer CPU times. Figure 7 shows the cumulative search distance over 100 rounds for each method, and Figure 8 shows the cumulative CPU time over 100 rounds.

**Figure 9:**
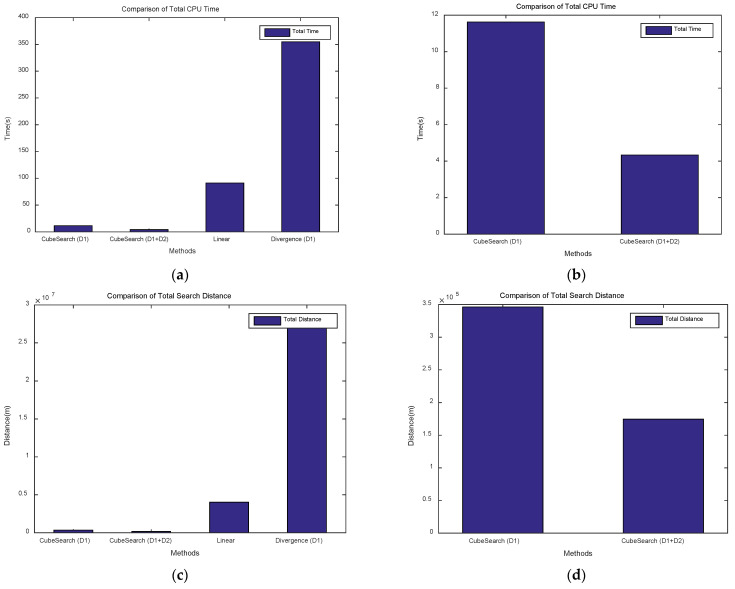
Comparison of Total CPU Time and Total Search Distance. (**a**) Four methods (Total CPU Time). (**b**) Two cube search methods (Total CPU Time). (**c**) Four methods (Total Search Distance). (**d**) Two Cube Search methods (Total Search Distance).

Figure 9a,b show the Total CPU Time for each method. Figure 9a presents the data for comparing between the four methods, showing that the CPU Time for the Linear and Divergence methods is relatively high. Figure 9b shows the CPU Time when the proposed cube-based search is performed using one and two drones. As shown in the figure, a cooperative search with two drones results in a shorter CPU Time than does a search with a single drone. This demonstrates that a cooperative search using two drones was more effective than a search using a single drone. As shown in the figure, the linear search method required approximately 21 times more CPU time than did the proposed method, whereas the divergence method required approximately 81 times more CPU time. Additionally, the method using a single drone consumed approximately 2.6 times more CPU time than did the cooperative search method using two drones.

Figure 9c,d show the Total Search Distance for each method. Figure 9c presents data for comparing the four methods, showing that the search distances for the Linear and Divergence methods were relatively high. Figure 9d shows the Search Distance when the proposed cube-based search is performed using one and two drones. As shown in the figure, a cooperative search with two drones results in a shorter Search Distance than does a search with a single drone. This demonstrates that a cooperative search using two drones is more effective than a single-drone search in terms of the search distance. As shown in the figure, the linear search method requires an approximately 23 times greater travel distance than does the proposed method, whereas the Divergence method requires an approximately 155 times greater travel distance. Additionally, the method using a single drone requires about two times the travel distance of the cooperative search method using two drones.

**Figure 10:**
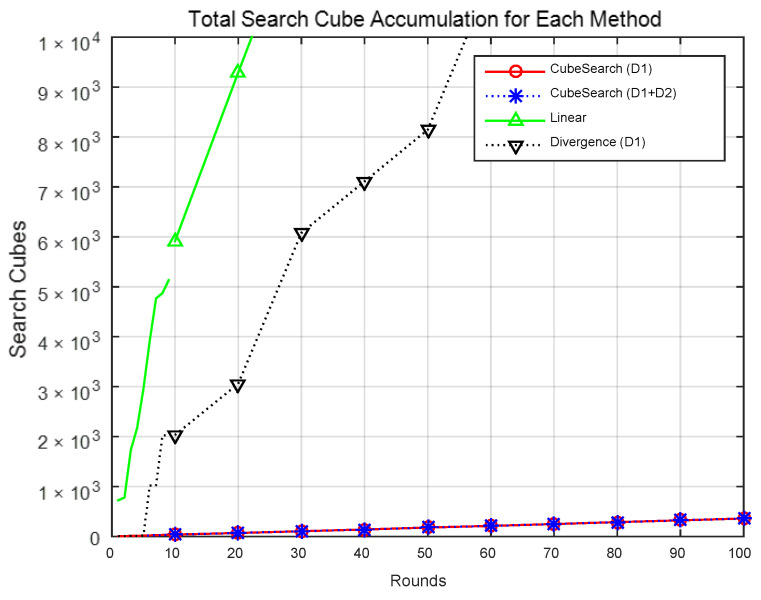
Total search cube accumulation for each method.

**Figure 11:**
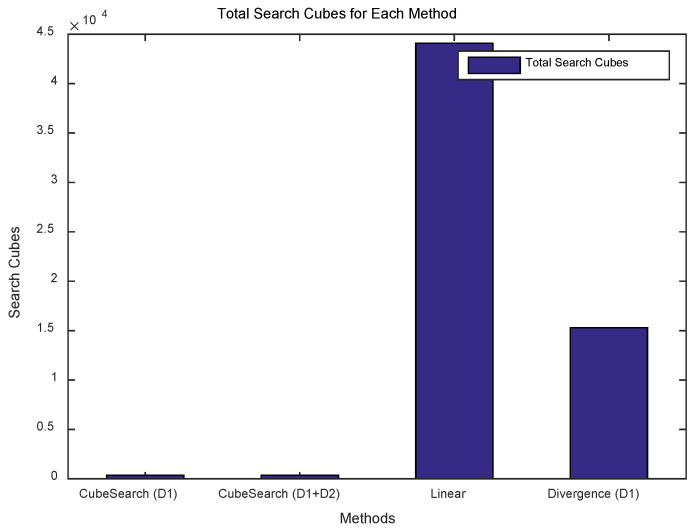
Total search cubes for each method.

Figure 10 shows the cumulative number of searched cubes for over 100 rounds for each method. Figure 11 shows the total number of searched cubes for each method. As shown in the figures, the proposed Cube Search method resulted in a lower number of searched cubes. That is, the linear search method explored approximately 122 times more cubes than did the proposed method, whereas the divergence method explored 42 times more cubes. The methods using one drone and two drones explored the same number of cubes. This is because the same algorithm was used, with the only difference being the number of drones used. This demonstrates that the proposed method was able to successfully complete a search while exploring fewer cubes, indicating a smaller search space.

### 4.2. Development of a Quadcopter for Air Pollution Detection

We designed an experimental drone to implement the proposed algorithm. As shown in Algorithm 1, the drones were developed in the form of quadcopters, with two drones created for cooperative exploration flights. Each drone was equipped with various air pollution sensors, including a PM sensor (particulate matter sensor) and CO/CO_2_ sensors, to ensure the accurate detection of atmospheric pollutants. In addition, GPS sensors were integrated into the drones to ensure precise navigation and collision avoidance. A Long-Range (LoRa) communication module was installed to communicate with the server. The specific hardware specifications of the developed drones are listed in Table 3.

**Figure 12:**
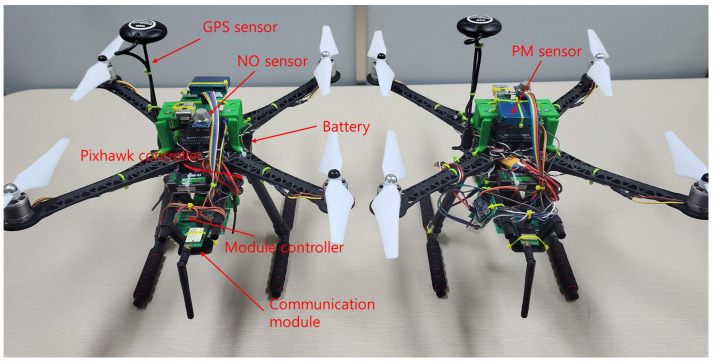
Drones developed for air pollution exploration through adaptive cooperation.

The drone developed for air pollution monitoring in this study is shown in Figure 12. The system controller was selected so as to have sufficient processing power to ensure that there were no delays or errors in communication. Air pollution sensors, including PM and CO sensors, were strategically positioned to minimize the effect of the propellers on the accuracy of the measurements. The communication module was positioned to optimize connectivity with the ground server, and the battery was positioned to maintain the center of gravity of the drone. Both drones were equipped with identical hardware components to ensure consistent performance. However, the software embedded in the controllers was assigned unique IDs to distinguish the two units during cooperative operations.

### 4.3. Experiments

**Figure 13:**
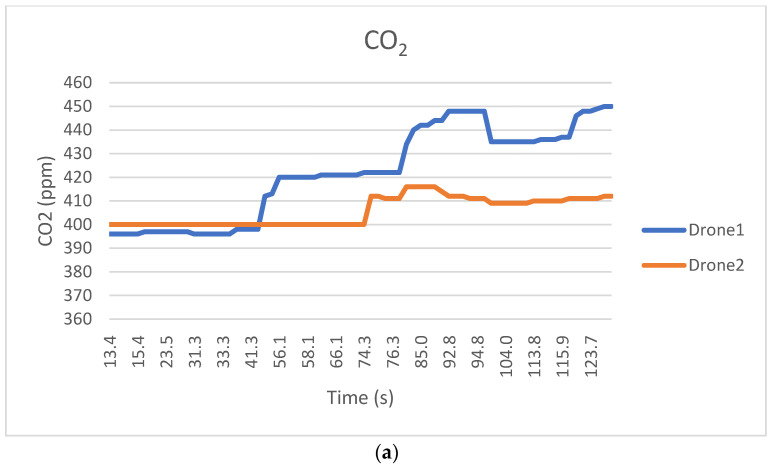

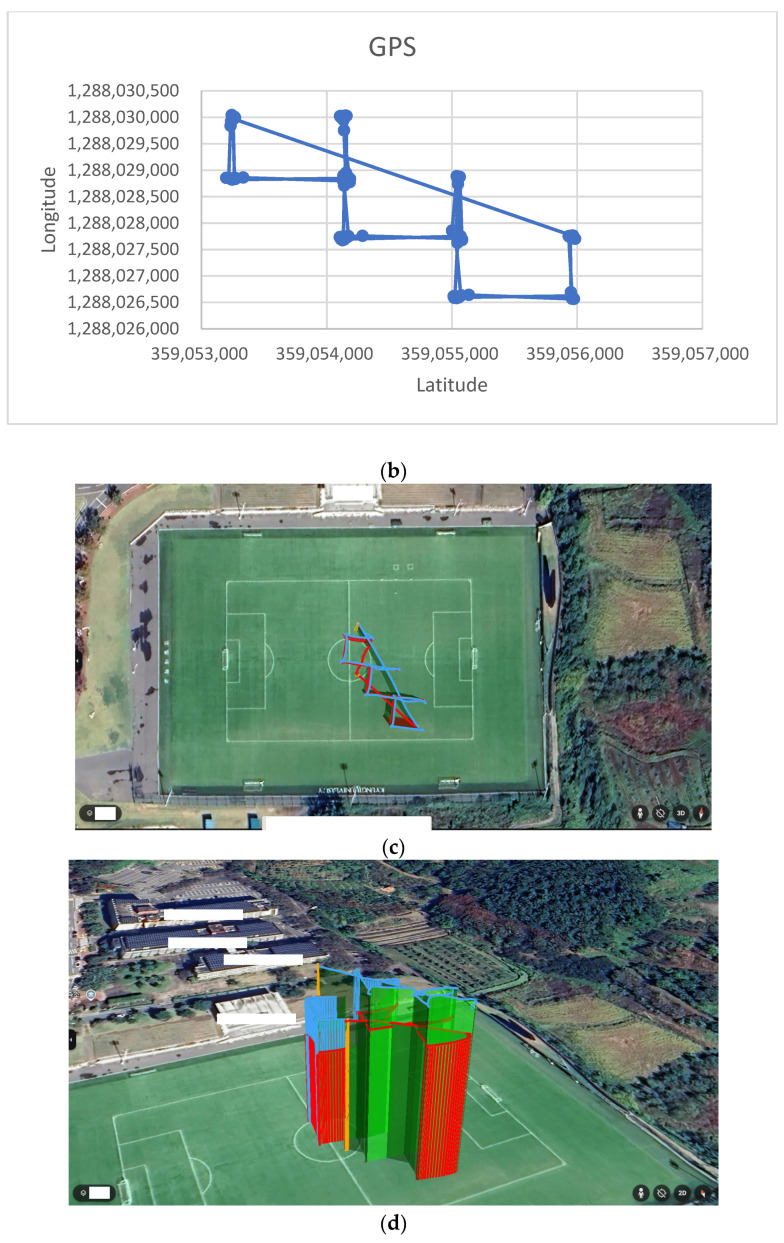
Collaborative exploration experiment using drones equipped with the proposed algorithm (site 1). (**a**) Exploring a CO_2_ threshold (450 ppm, site 1). (**b**) Changes in drone position during exploration (site 1). (**c**) Exploration trajectory tracking (2D, site 1, Blue: Drone 1, Red: Drone 2). (**d**) Exploration trajectory tracking (3D, site 1, Blue: Drone 1, Red: Drone 2, Green: Altitude, Yellow: Descent).

The proposed 3D cube-based adaptive cooperative search algorithm was tested in a real-world environment by equipping a developed quadcopter drone with the algorithm. The experimental conditions are listed in Table 4. Real-world experiments were conducted at two different sites: a university campus playground in a relatively urban area and an outdoor playground that was relatively distant from the buildings. Because the maximum level of air pollution within the search area was unknown, a threshold value was set, and the search ended when a level exceeding the threshold was detected. If the threshold level was not detected, the search could become out of control; therefore, the Maximum Search Altitude and Maximum Search Width were set to ensure that the search was conducted within a limited space. From the ground, the two drones began their search approximately 30 m apart under the command of the server. All the controls for cooperative drone flights were autonomous. The search ended when the air pollution level that was set as the threshold was detected under the cooperative flight of the two drones, and the drones returned to their starting point. Figure 13 shows the air pollution search results for the two cooperating drones at Site 1. Figure 13a shows the process of searching for a CO_2_ threshold of 450 ppm; Figure 13b displays the latitude and longitude of the drones during the search; Figure 13c tracks the drones’ trajectory against a real-world background; and Figure 13d shows the trajectory of the drones in 3D.

**Figure 14:**
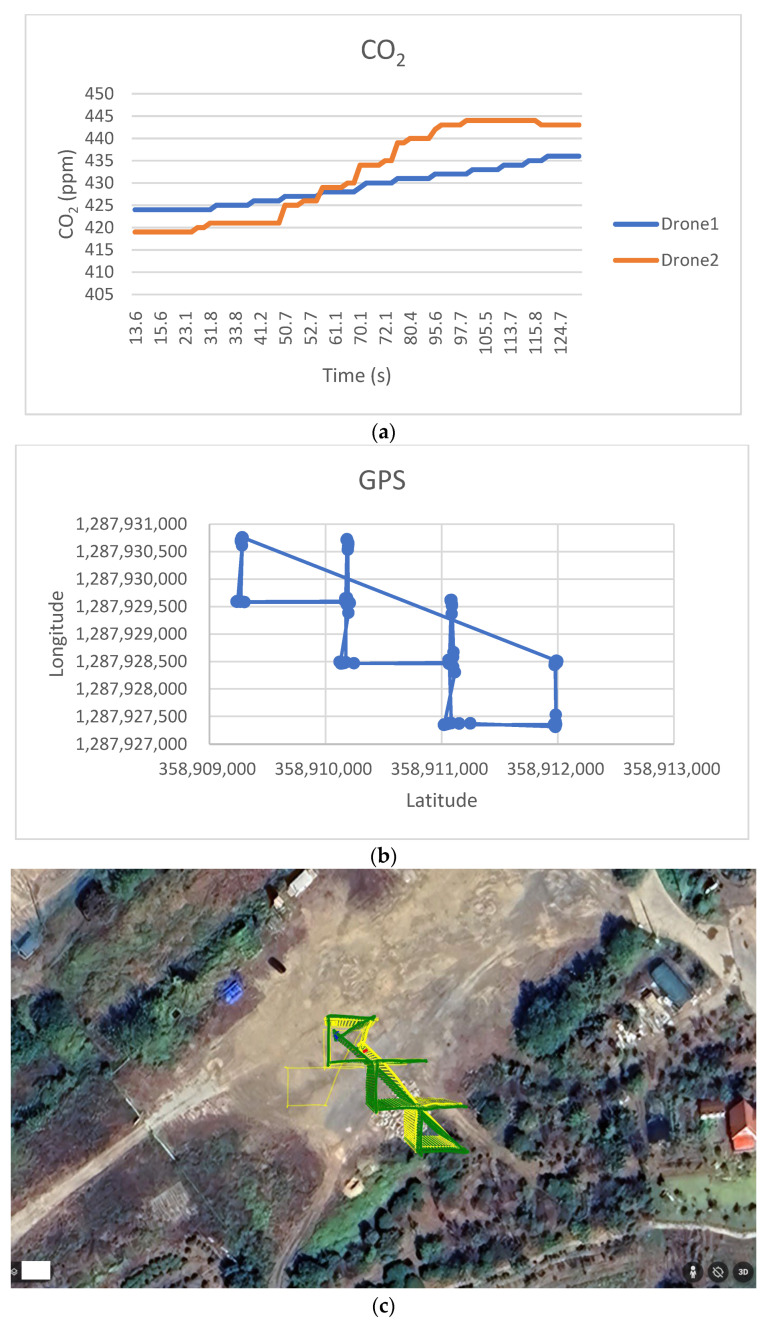

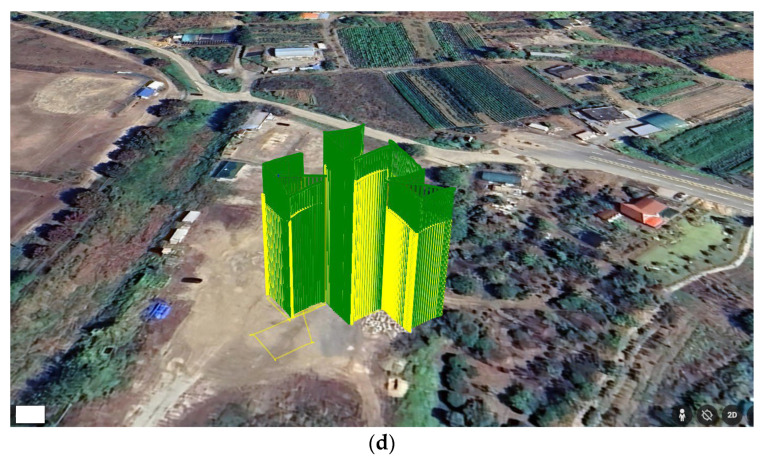
Collaborative exploration experiment using drones equipped with the proposed algorithm (site 2). (**a**) Exploring a CO_2_ threshold (444 ppm, site 2). (**b**) Changes in drone position during exploration (site 2). (**c**) Exploration trajectory tracking (2D, site 2, Green: Drone 1, Yellow: Drone 2). (**d**) Exploration trajectory tracking (3D, site 2, Green: Drone 1, Yellow: Drone 2).

In this experiment, the length of one side of the cubic search space for air pollution detection was set to 10 m, as shown in Table 4. Within this cubic search space, one drone was assigned to explore the upper portion of the cube, while the other drone explored the lower portion. As a result of this spatial separation, the CO_2_ concentration values collected by the two drones differed, as shown in Figure 14a. During the air pollution search, if either of the two drones detects a concentration above the threshold, the search is considered successfully completed.

Figure 14 shows the air pollution search results for a cooperative search by the two drones at Site 2. Figure 14a shows the process of searching for a CO_2_ threshold of 444 ppm; Figure 14b displays the latitude and longitude information for the drones during the search; Figure 14c tracks the drones’ trajectory against the real-world background; and Figure 14d shows the trajectory of the drones in 3D.

## 5. Discussion

Through simulations, it was demonstrated that the proposed search method outperformed the linear search method and that the cooperative search method using two drones was superior to the method using a single drone. Furthermore, the effectiveness of the proposed algorithm was validated through real-world experiments using quadcopter drones equipped with the algorithm. In this section, we discuss several key points related to the design of the algorithm, simulation process, development of the quadcopter, and execution of real-world experiments.

First, we discuss the uncertainty in the distribution of air pollution gases within the search space and the cube-based search method: Owing to the uncertainty in the distribution of air pollution gases within the search space, we selected a cube-based search method to allow for more precise exploration. There may be multiple cubic spaces that contain pollution levels that exceed the threshold; however, the proposed algorithm targets the space that was first explored according to a cube-based search. The algorithm can be modified and extended to explore regions having pollution levels above a threshold. Although simulations can use the cube with the highest pollution level to conduct the search, the highest pollution value in real environments is unknown, thus it is more reasonable to terminate the search once a value exceeding the threshold is detected.

Second, we discuss drone hardware development issues: In terms of drone hardware development, issues such as sensor sensitivity and accuracy, sensor attachment location, battery operation time, and drone altitude control must be considered. The sensors used in this study were relatively standard and inexpensive, as listed in Table 3. Because the air pollution levels in the cubic units of the search space did not vary greatly, sensors having higher performance are needed to accurately identify pollution locations. Regarding sensor placement, studies such as that of Araujo et al. [37] discussed optimal sensor positioning to minimize the effects of propeller and drone flights. The selection of an appropriate battery is a critical factor in drone exploration. This choice is closely tied to the performance of the propeller motors, as well as the search time and depth. High-capacity batteries are needed to cover a wider area and to supply powerful motors; however, considerations such as drone weight and reduced exploration time must also be considered.

Third, we discuss issues related to the drone cooperative search: The proposed 3D cube-based adaptive cooperative search algorithm was designed for two drones to cover a cube-shaped search space and explore it cooperatively. This algorithm could be extended to allow more drones to participate in searches. However, additional considerations include the cost of adding more drones, the potential for drone collisions during movement within the cube space, and higher complexity of the algorithm.

Fourth, this study discusses the scalability of the proposed algorithm and the developed drone system. To evaluate their performance, experiments were conducted in two locations, described in Table 4: a university sports field and a general outdoor space. While the current experiments were conducted in limited environments due to safety concerns and legal regulations related to drone flight, we believe the system can be extended to various real-world environments such as urban areas or industrial zones, where the likelihood of air pollution is higher. Expanding experiments to such environments would further enhance the algorithm’s applicability and potential for commercialization.

Fifth, we acknowledge a limitation related to the impact of wind speed on the performance of the proposed algorithm and the developed quadcopter drone. This study did not consider the effect of varying wind conditions on air pollution measurements, as the focus was placed more on identifying pollution-prone areas than on evaluating environmental variables like wind. However, we expect that wind speed—both low and high—could affect measurement accuracy. Thus, we plan to address this issue in future studies as a separate research topic.

Sixth, one of the key strengths that differentiates this study from previous works is its integrated and application-oriented approach. As discussed in Section 2 (Related Works), there are relatively few studies exploring the use of drones for air pollution detection, and even fewer that propose practical, deployable systems. In this study, we not only propose a novel cube-based spatial search algorithm for air pollution monitoring but also develop a quadcopter drone capable of running the algorithm in real-world scenarios. The algorithm was implemented in software and embedded in the drone, and its effectiveness was verified through field experiments. This end-to-end approach—from algorithm design to hardware development and real-environment testing—significantly enhances the practical applicability of our method.

## 6. Conclusions

In this study, a 3D cube-based adaptive cooperative search algorithm for air pollution exploration was proposed, and its efficiency was validated through simulations and experiments. The simulation results showed that the proposed method achieved a target detection rate that was approximately 15% higher than that of Divergence search. In terms of CPU time, the linear search consumed 21-fold more time than did the proposed method, whereas the Divergence method required 81-fold more time. Regarding the search distance, the linear search required 23 times greater movement distance, and the Divergence method required 155 times greater distance than did the proposed method. Additionally, the linear search explored 122 times more cubes than did the proposed method over 100 rounds, whereas the Divergence method explored 42 times more cubes than did the proposed method.

When the single-drone search method was compared with the cooperative search method using two drones under the same algorithm, the single-drone method required approximately 2.6 times more CPU time than did the two-drone cooperative search. Similarly, in terms of search distance, the single-drone method required approximately twice the search distance than did the cooperative search. These results demonstrate that the proposed cube-based cooperative search using multiple drones is more efficient than are other methods.

To validate the effectiveness of the proposed algorithm, two quadcopter-based drones were developed, as shown in Figure 12, and the proposed algorithm was implemented. Real-world air pollution exploration experiments were conducted at two locations: a university sports field in an urban area and an open outdoor space. The experimental results confirmed that the drones equipped with the proposed algorithm successfully completed their mission by autonomously searching for areas that exceeded the threshold pollution level, in accordance with the algorithm. Therefore, the proposed algorithm is applicable to real-world air pollution exploration environments.

Therefore, the main outcomes of this study can be summarized as follows:

- Development and validation of a cube-based multi-drone cooperative search algorithm for air pollution detection: A cooperative drone search algorithm based on a 3D cube structure was proposed to efficiently detect air pollution in three-dimensional space. Numerical simulations demonstrated the efficiency of the proposed algorithm. Specifically, in terms of CPU time, the proposed algorithm consumed approximately 21 times less CPU time than the linear search method and 2.6 times less than the single-drone search. In terms of search distance, the proposed algorithm required approximately 23 times less distance than the linear search and about twice less than the single-drone search.
- Development of drone hardware, implementation of the proposed algorithm, and validation through real-world experiments: A quadcopter-type drone was developed to implement and verify the proposed algorithm. Experiments were conducted in real-world environments, as described in Table 4. The results confirmed the effectiveness of both the developed drone and the proposed algorithm. In particular, by setting a threshold value for CO_2_ concentration during air pollution search missions, the drone was able to autonomously complete its mission and successfully detect concentrations above the threshold, demonstrating the feasibility of the system.

Based on the research process and conclusions, the significance of this study can be summarized as follows.

First, a novel and practically applicable algorithm for air pollution exploration was proposed. A search method was developed to explore the vertices of cube-shaped spaces, identify the highest pollution concentration point, and connect to new cube search spaces accordingly. This approach allowed for a more detailed exploration of a given search space and enabled a rational expansion of the search area. The proposed search method was validated through simulations and real-world experiments, which demonstrated its efficiency and effectiveness.

Second, a well-connected and comprehensive study was conducted to address air pollution. Initially, an investigation of the search space led to the development of a cube-based search method. Accordingly, a cooperative search algorithm that would be suitable for multiple drones was designed, and its efficiency was verified through simulations. Subsequently, drone hardware was developed for real-world applications, and the designed algorithm was implemented in drones, which was followed by the execution of real-world experiments. This continuous development and experimentation process adds value to the research model for drone-based air pollution exploration. Before this study was conducted, a literature review on drone-based target detection, tracking, and monitoring was conducted. This study is expected to serve as an important reference for future studies on drone-assisted target exploration, including air pollution detection.

Future research will proceed in the following directions. First, to overcome the limitations discussed in the Discussion section, experiments will be conducted in various environments and locations to observe how air pollution measurements change depending on environmental conditions and geographical factors. In particular, we aim to investigate how variables such as wind influence and cube size affect pollutant concentration measurements. Second, the proposed cube-shaped search space will be extended to include various geometric configurations in order to explore how the shape of the search space impacts exploration efficiency. Future work will involve designing and comparing algorithms that operate in alternative search spaces, such as cones, pyramids, and polyhedrons, with the goal of identifying the most effective spatial structure for environmental exploration.

## Figures and Tables

**Table 2:** Simulation environment.

Category	Value
Size of search area	10 × 10 × 10 cells
Cell size	1 × 1 × 1 m
Threshold value	0.9
Target (maximum) value	1.0
Number of targets	1 (random)
Number of UAVs	1 and 2
Limitations on navigation rounds	1000

**Table 3:** Drone hardware components and specifications.

Components	Specifications
System controller	Raspberry Pi Pico, Dual-Core Arm Cortex-M0+, 133Mhz
Communication module	LoRa E22-900M22S
PM sensor	pms7003
CO, CO_2_ sensor	MTP40-F NDIR CO_2_ sensor
Frame	TAROT S500 Quad-Copter frame, Diameter: 500 mm, Height: 288 mm
Motors	S2312-920KV Motor, 23 × 12 mm
Propeller	9 × 4.5 inch Self-Lock Propeller, Universal Rype
Flight controller	Pixhawk 2.4.8 32 bit
GPS sensor	UBlox M8N GPS
Battery	PT-B5200N-FX50 (14.8V, 4S1P, 50C+)

**Table 4:** Experimental environment.

Components	Specifications
Number of Drones	2 quadcopter-type drones
Experimental Site	Site 1. University playground Site 2. Outdoor Open Space
Initial Search Altitude	15 m
1 Unit	10 × 10 × 10 m Cube Type
Number of Explorations in One Cube	2 times
Maximum Search Altitude	60 m
Maximum Search Width	100 m

## Data Availability

Data are contained within the article.

## Abbreviations

The following abbreviations are used in this manuscript:

CTO	Collaborative trajectory optimization
FHM	Forest health monitoring
GUI	Graphical user interface
IoT	Internet of Things
LoRa	Long-Range
PSO	Particle swarm optimization
PM	Particulate matter
UAV	Unmanned aerial vehicle
UGV	Unmanned ground vehicle
WSN	Wireless sensor network
YOLO	You Only Look Once
